# Setting Up a Bioluminescence Resonance Energy Transfer High throughput Screening Assay to Search for Protein/Protein Interaction Inhibitors in Mammalian Cells

**DOI:** 10.3389/fendo.2012.00100

**Published:** 2012-09-11

**Authors:** Cyril Couturier, Benoit Deprez

**Affiliations:** ^1^Univ Lille Nord de FranceLille, France; ^2^INSERM U761, Biostructures and Drug DiscoveryLille, France; ^3^Université du Droit et de la Santé de LilleLille, France; ^4^Institut Pasteur LilleLille, France; ^5^Pôle de Recherche Interdisciplinaire sur le MédicamentLille, France

**Keywords:** BRET, PPI, P2I2, RET, HTS, screening assay, inhibitor compound, modulator compound

## Abstract

Each step of the cell life and its response or adaptation to its environment are mediated by a network of protein/protein interactions termed “interactome.” Our knowledge of this network keeps growing due to the development of sensitive techniques devoted to study these interactions. The bioluminescence resonance energy transfer (BRET) technique was primarily developed to allow the dynamic monitoring of protein/protein interactions (PPI) in living cells, and has widely been used to study receptor activation by intra- or extra-molecular conformational changes within receptors and activated complexes in mammal cells. Some interactions are described as crucial in human pathological processes, and a new class of drugs targeting them has recently emerged. The BRET method is well suited to identify inhibitors of PPI and here is described why and how to set up and optimize a high throughput screening assay based on BRET to search for such inhibitory compounds. The different parameters to take into account when developing such BRET assays in mammal cells are reviewed to give general guidelines: considerations on the targeted interaction, choice of BRET version, inducibility of the interaction, kinetic of the monitored interaction, and of the BRET reading, influence of substrate concentration, number of cells and medium composition used on the *Z*′ factor, and expected interferences from colored or fluorescent compounds.

## Introduction

Protein/protein interactions (PPI) govern all key events in a cell life, from division, to adaption or response to extracellular signals leading to biological effects. However, this view was not so obvious in the past, as convincing examples demonstrating such phenomena were exceptional and hard to achieve. In the last decade, numerous methods with increasing sensitivities and potencies have been developed, allowing the monitoring of those interactions (Xu et al., 1999; Tavernier et al., 2002; Chan, 2004; Brovko and Griffiths, 2007; Michnick et al., 2007; Ventura, 2011; Hamdi and Colas, 2012). Evolution of such methods has allowed the dynamic detection of PPI in living cells (Xu et al., 1999; Coulon et al., 2008; Lee et al., 2010; Quiñones et al., 2012) and nowadays in whole living organisms (Subramanian et al., 2004; Audet et al., 2010). Following this evolution scheme, PPI pathways have been deciphered and furthermore organized in higher protein networks ranging from PPI taking place in molecular complexes, to entire organelles and to whole organisms (Coulon et al., 2008; Chautard et al., 2009; Jaeger and Aloy, 2012). Our current knowledge of these PPI networks has further increased in recent years with the emerging idea that more than PPI networks themselves, the biological context in which they occur is important. System wide analyses of PPI crossing genetic data or pathological states of the cells from which they were generated have been performed and led to new data pointing out the changes in PPI networks in some human pathology (Bader et al., 2008). Deciphering that a fine PPI change can lead to a drastic PPI network modification was the bases of a pathological state, has opened new views for drug discovery. Applying this concept by using the current knowledge of protein interaction network modification in glioblastoma cancer cells, a recent study allowed the successful screening of inhibitory peptide disrupting PIKE-A/Akt and their capacity to inhibit the proliferation of these cells (Qi et al., 2012). Attempts to gain exhaustive interactome taking place in diseases have became common. These growing data demonstrate that most proteins interact with more than one partner (Krause et al., 2004) and lead to better drug target choosing. Indeed the deciphering of deregulated or key interactions in diseases crossed with interactions involved in the less pathways allows to minimize or avoid unexpected side effects (Chen et al., 2012).

To search for inhibitors of PPI, the same methods used to detect the interactions can be used. The need for robust and high throughput screening (HTS) compatible method, when performing screening assays, has lead to the preferential use of techniques such as yeast two hybrid and derivatives (Hamdi and Colas, 2012), Fluorescence polarization (Smith and Eremin, 2008), MAPPIT (Lievens et al., 2011); and protein complementation assay (Morell et al., 2009; Michelini et al., 2010). Other methods based on resonance energy transfer (RET) to monitor PPI, offers great advantages as they allow full length proteins dynamic interaction monitoring in intact cellular contexts and are applicable to HTS (De, 2011). In this review, the use of RET and more advantageously PPI inhibitors (P2I2) bioluminescence resonance energy transfer (BRET)-based screening assays in mammalian cells will be developed.

## The Different RET Methods

**Figure 1:**
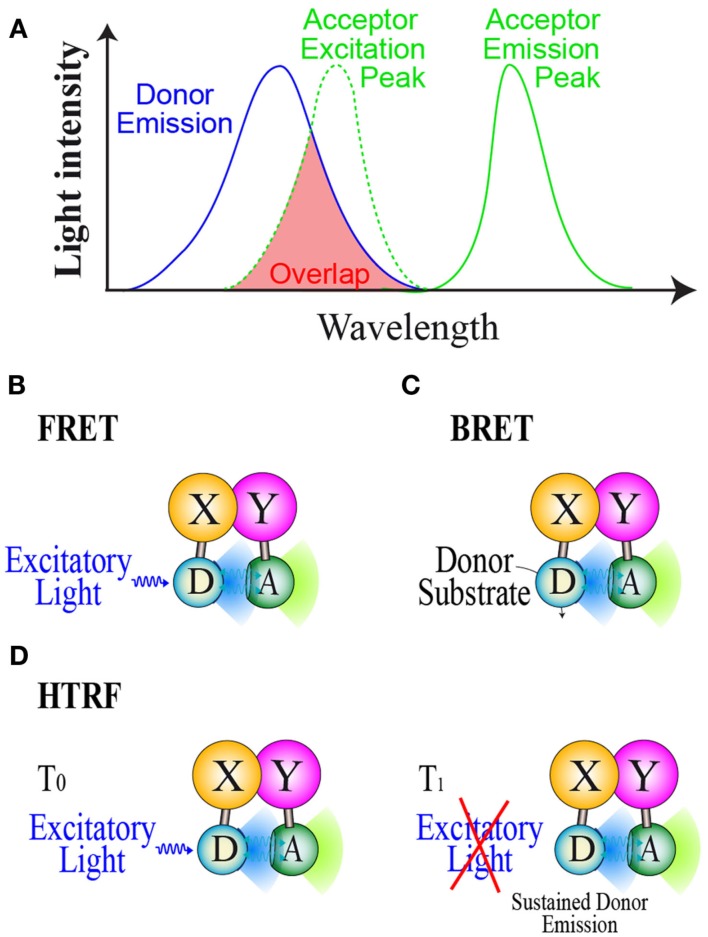
**Resonance energy transfer methods. **(A)**** Basic properties of donor/acceptor compatible couple in order to gain RET. Principles of **(B)**: the FRET method, **(C)**: The BRET method, and **(D)**: the HTRF method. D, Donor; A, Acceptor; S, Substrate.

To date, three main RET methods have been developed and used in drug screening assays: FRET (Forster Resonance Energy transfer), BRET and HTRF (Homogeneous Time Resolved fluorescence). All RET methods are based on the use of compatible energy donor and acceptor couples allowing RET to take place when donor and acceptor are in close proximity (<10 nm). To be a compatible couple, the energy donor emission wavelength has to overlap the energy acceptor excitation one in order to gain energy transfer (Figure 1A). The energy donor and acceptor are each linked to one of the interacting partners and resonance can occur if the two partners interact and close the donor and acceptor by a distance less than 10 nm. In the FRET method (Figure 1B), donor and acceptor are both fluorophores and a proper excitatory light is needed to promote donor emission (Fruhwirth et al., 2011). In FRET cellular screening assays, donor and acceptor are two fluorescent proteins each genetically fused to one of the interacting partners. In the BRET method (Figure 1C), the energy donor is a bioluminescent enzyme, converting its substrate into light emission able to promote RET with a compatible fluorescent acceptor (Pfleger and Eidne, 2006; Bacart et al., 2008). For live cell screening purpose, BRET assays involve genetically engineered fusion protein of the studied partners respectively with the donor and acceptor. HTRF is an enhanced FRET derivative method which circumvents the major FRET problem due to simultaneous excitation of acceptor by donor excitatory light. This method is based on energy transfer monitoring in a time resolved manner (Degorce et al., 2009). Indeed the donor used is a fluorescent molecule able to emit light for a short time period after the excitatory light has been turned off (Figure 1D). This last property allows the monitoring of energy transfer to a compatible acceptor once the excitatory light is switched off.

All these RET methods have several advantages over the other methods to monitor PPI, that make them the best suited method to detect PPI in mammalian cells. FRET, HTRF, and BRET are homogenous assays as the energy transfer signal is only emitted from the interacting partners, and then, no artifact prone washing steps are required before reading. Each of these methods has its advantages and limits that make them best suited methods in certain fields. In P2I2 live cell screening assays BRET present several advantages over other RET methods.

## Why Choosing BRET to Screen for PPI Inhibitors?

Classical FRET and BRET screening assays have a subsequent advantage over HTRF as they mostly rely on genetically fused energy donor and acceptor proteins respectively to both partners implicated in the monitored interaction. Using such fusion proteins can however be a disadvantage as fusion can promote steric hindrance hindering wild type interactions. On the other hand, HTRF is able to monitor unmodified protein interactions but involves a latter step of protein labeling with antibodies or chemical linkage (Degorce et al., 2009) which lower its interest in live cell P2I2 HTS assays. BRET shows several advantages over FRET (Boute et al., 2002): first, the excitation of the donor fluorophore by monochromatic light in FRET also lead to the concomitant excitation of the acceptor then hardening the results interpretation; second, this excitatory light promote photobleaching of the donor and cell autofluorescence; and third, BRET signal/noise ratio has been shown to be 10-fold higher than FRET thus allowing the use of 40-fold less amount of protein to reach the same signal level than FRET (Arai et al., 2001). This last parameter is important for screening P2I2 as over-expression of proteins (excess of the monitored complex) might titer a potential active molecule leading to its inability to promote the expected decreased in signal. Indeed, BRET superiority was shown by its ability to monitor PPI using endogenous level of protein expression (Couturier and Jockers, 2003; Pfleger and Eidne, 2003) and its consequent application to various live cell screening assays (Pfleger et al., 2007; Bacart et al., 2008; Kocan and Pfleger, 2011). Finally, using this method to screen for P2I2 is further supported as BRET is prone to disruption or modulation by co-expression of untagged interacting partner (Bacart et al., 2008; Ayoub and Pfleger, 2010; Kulahin et al., 2011) and by incubation with inhibitory peptides (Granier et al., 2004; Harikumar et al., 2006; Jarry et al., 2010) or inhibitory chemical compounds (Mazars and Fåhraeus, 2010; Corbel et al., 2011).

## For Which Kind of Target Interaction Can the BRET be Chosen?

**Figure 2:**
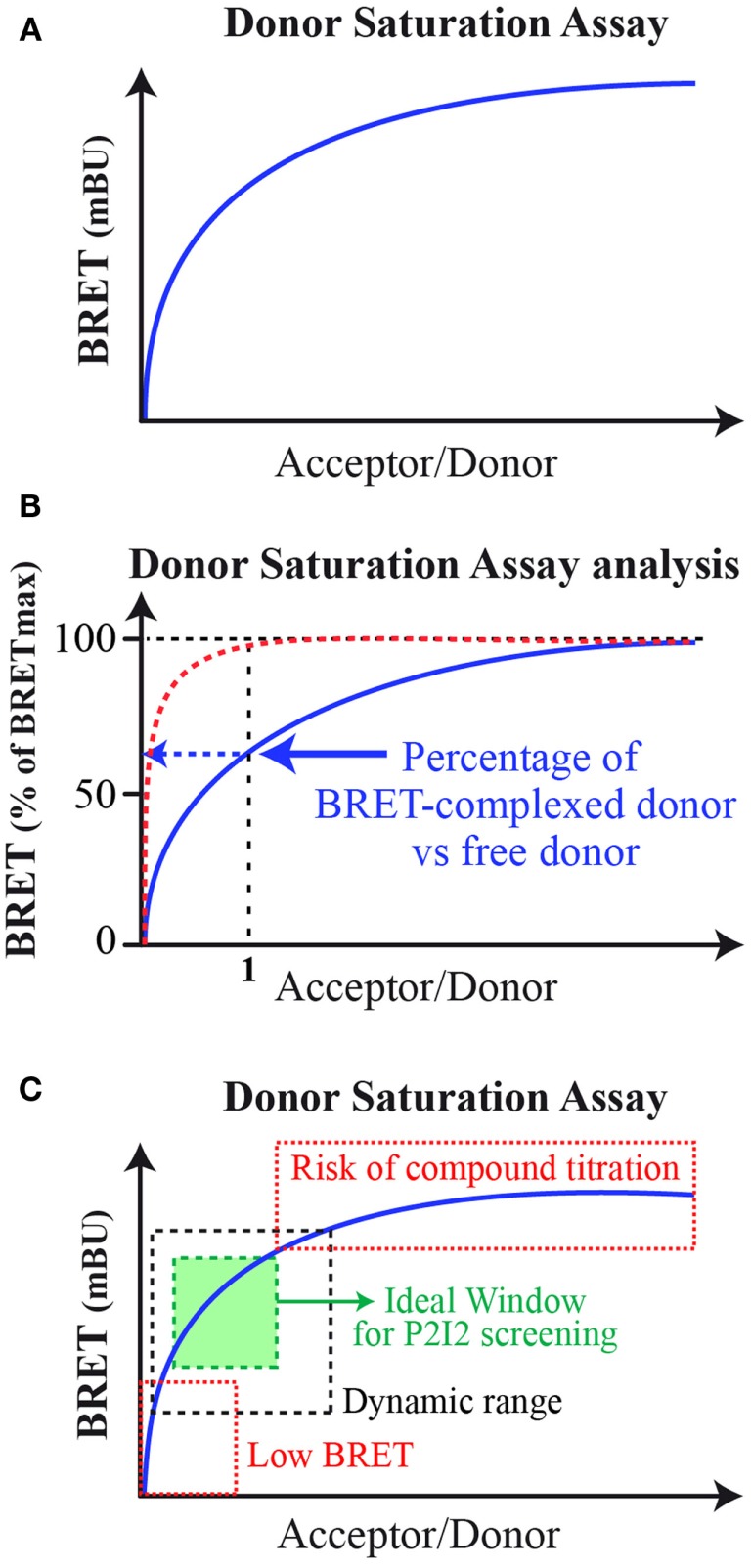
**Bioluminescence resonance energy transfer donor saturation assay**. **(A)** Basic donor saturation assay expressed as milliBRET unit (mBU); **(B)** Donor Saturation Analysis for dimeric complexes formation: in red, theoretical curve if 100% donor and acceptor interact with each other at a 1/1 molar ratio. In blue, the common DSA curves obtained showing lower percentage of donor/acceptor complexes in cells. **(C)** Donor saturation assay for analysis to set up a P2I2 BRET-based screening assay. In hatched black, the dynamic windows of BRET monitoring. In hatched red, the two areas of the DSA curve to avoid. In green, the ideal window to be chosen when setting up a P2I2 BRET-based screening assay.

The BRET method has already been applied to monitor interaction between various kinds of proteins partners and in various cellular components (Bacart et al., 2008; Alvarez-Curto et al., 2010). This range from two soluble proteins, two transmembraneous ones, one transmembraneous, and one soluble, with interactions taking place in cytoplasm, nucleus, and cytoplasmic or internal membranes (Coulon et al., 2008; Guan et al., 2009; Bacart et al., 2010). Indeed BRET is able to monitor all kinds of interaction, however, certain concerns have to be taken into account when designing P2I2 BRET-based assays. First, the BRET signal is dependent on the donor/acceptor ratio as described by the well-known donor saturation assay (DSA; Mercier et al., 2002; Bacart et al., 2008; Ayoub and Pfleger, 2010; Figure 2A). The DSA leaded to further analyze of the BRET signal and demonstrated that the maximal BRET intensity is dependent on the ratio of energy donor interacting with an energy acceptor versus free energy donor present in the cell (Couturier and Jockers, 2003; Ayoub et al., 2004; Figure 2B). Indeed at equimolar ratio, if all donors and acceptors molecules interact together, a maximal BRET would be raised. However, this is rarely the case and free donors molecules (or interacting with other but non-acceptor-tagged proteins) lead to decrease this maximal BRET value. Given that, in order to gain the higher BRET signal, the acceptor fusion protein would be highly expressed compared to the energy donor to lower the free donor proportion. However, to ensure the monitoring of active compounds effects, the titration of the compound by excess acceptor has to be avoided. In order to prevent this phenomenon, the level of expression of both partners would result in an ideal window leading to high BRET signal still located in the dynamic range of DSA curves (Figure 2C).

Furthermore, this last parameter will guide the choice for the design of the fusion proteins. As the proportion of free donor will lead to decrease the BRET signal, it has to be the lowest and the choice to fuse it to a *X* or *Y* protein will be the global ratio of *X*/*Y* complexes versus *X* or *Y* that are free or engaged in other complexes than the one studied.

Bioluminescence resonance energy transfer is also well suited to monitor transitory interaction but with the same restriction: when performing the reading, the BRET signal will depend on the percentage of donor/acceptor complexes versus the donor alone and would be hard to monitor if this percentage is low. Some modifications can enhance the monitoring of such interaction like substrate trapping strategy that disables the substrate/enzyme dissociation (Boute et al., 2003; Issad et al., 2005; Boubekeur et al., 2011).

## Which BRET Version to Chose?

To screen for P2I2, compound titration by excess reporter amount has to be avoided. For *in vitro* interaction methods, setting up the protein quantities to use is easily done, however this is harder to achieve for live mammalian cell BRET-based assays. Indeed, choosing the most sensible and most compatible with HTS over the different BRET versions available seems to be the only way to gain the necessary highest readout. This choice became difficult nowadays as several BRET methods based on different substrates and different compatibles donor/acceptor couples have been developed (Bacart et al., 2008; De et al., 2009; Lohse et al., 2012).

### BRET1

Original BRET1-based on the Rluc/YFP couple showed low signal (Xu et al., 1999) hindering its use in HTS. Higher signals were obtained using mutants or new cloned acceptors such as YFP Topaz, YFP citrine, YFP Venus, YPet, or the Renilla-GFP (R-GFP; Bacart et al., 2008; Molinari et al., 2008; Kamal et al., 2009; Ayoub and Pfleger, 2010). YFP Venus was used to demonstrate the feasibility of a BRET1 HTS assay in CCR5 ligands screening (Hamdan et al., 2005). The BRET1 readout signal was also enhanced by the concomitant use of these acceptors with mutants of Rluc or other luciferases. Rluc2 or Rluc8, mutants of Rluc with higher stability and quantum yield (Loening et al., 2006), greatly increased BRET1 signal (Kocan et al., 2008; Kamal et al., 2009; Schelshorn et al., 2012). Recently, BRET1 was used to develop two P2I2 screening assays (Mazars and Fåhraeus, 2010; Corbel et al., 2011).

BRET1 has also been achieved using Gaussia Luciferase (Gluc). Gluc is a smaller and brighter luciferase known to date and was cloned from a marine copepod (Tannous et al., 2005; Welsh et al., 2009). It shares some spectral properties with Rluc and has been recently used in BRET1 assays (Li et al., 2012).

BRET1 method using quantum dot (Qdot) as energy acceptors has also been reported these past few years. These photostable fluorescent nanoparticles are excitable at 480 nm and have a size dependent emission wavelength tunable to the overall rainbow colors (Weng and Ren, 2006). Qdot BRET-based assay have first shown energy transfer efficiency (So et al., 2006) and *in vitro* protease assays have been later developed (Xia et al., 2008; Kim and Kim, 2012). However, the coupling to proteins (Algar et al., 2010) and the cellular toxicity (Soenen et al., 2012) of Qdot are still an obstacle to their use in live mammalian cell for PPI monitoring.

### BRET2

Bioluminescence resonance energy transfer 2 method was developed by Packard Biosciences by increasing the separation of the two emitted wavelength to circumvent the poor signal/noise ratio of BRET1. This enhancement relies on the concomitant use of coelenterazine 400a (or deep blue C), a coelenterazine derivative that forces the Rluc emission to a 397 nm peak, and the compatible energy acceptor GFP2 (a mutant of aequorea GFP; Ramsay et al., 2002). BRET2 has been successfully used for ligands screening (Vrecl et al., 2004; Elster et al., 2007), and virus protease inhibitors screening (Hu et al., 2005; Oka et al., 2011). However, BRET2 has suffered from a weak and short lasting light emission that greatly limited its use to develop P2I2 BRET-based HTS assays. Indeed, high expression of BRET partners is necessary to ensure signal recording. BRET2-based PPI assay using Rluc2 or Rluc8 have shown enhanced BRET dynamic range and kinetic of the reading up to 1 h (De et al., 2007; Kocan et al., 2008; Dacres et al., 2009, 2012; Kulahin et al., 2012). However, BRET2 has not been used in P2I2 screening assays yet and its use in this field would still need to be demonstrated.

### BRET3

A BRET3 method using firefly luciferase (Fluc) and dsRed or Cy3 as compatible acceptor has been developed (Arai et al., 2002; Yamakawa et al., 2002). However, the huge overlap of donor/acceptor emission peaks of this method leads to extremely low signal to noise ratio that impaired its application to really study protein/protein interactions. A better proof of concept was gained by the use of mOrange as acceptor that allowed PPI monitoring in live cells and animals (De et al., 2009). More recently, new analogs of luciferin (the firefly substrate), leading to different spectral properties of the emitted light, were synthesized and showed their efficiency in BRET3 experiments (Takakura et al., 2010, 2011). One of these, coumarylaminoluciferin allowed a mutant of Fluc to emit light compatible with the use of YFP as acceptor (Takakura et al., 2010) and may promote advances of the BRET3 version by using the various YFP variants developed for BRET1. At this stage of development, BRET3 has not been yet demonstrated to be a valuable method to screen for P2I2.

### Future BRET enhancements

Although major advances have already been made since the 1999s BRET version, further improvements of BRET methods are still expected. As described above, the BRET enhancements were based on the use of variants of luciferases or fluorescent acceptors, coupled to the concomitant use of modified substrates. New improvement of know luciferases are on the way and would probably lead to new BRET advances. A systematic pairing of luciferases with compatible substrates have highlighted best couples: Rluc/enduren and Gluc/native coelenterazine h are 8- to 15-folds brighter than the princeps BRET1 (Kimura et al., 2010). Another study sorted mutants of Gluc with a up to sixfold enhancement in light emission and a 10-fold prolonged bioluminescence than native Gluc which was already the brighter luciferase (Kim et al., 2011). *Vargula* luciferase (Vluc) shares quite the same spectral properties than Rluc and has been applied to BRET1 (Otsuji et al., 2004). *Metridia pacifica* luciferase 1 (MPluc1) and *Metridia longa* luciferase (Mluc) or its mutants emits in the 450–500 nm range and have thus potential to be used in BRET assays in the future (Takenaka et al., 2008; Kim et al., 2011; Markova et al., 2012). Recently, Nanoluc^™^, a new deep-sea shrimp evolved luciferase has been introduced by Promega (Hall et al., 2012). This 171 amino acids (19 kDa) ATP independent glow-type luciferase using furimazine as substrate is announced to have more than 100-fold higher luciferase activity than Rluc or FLuc. Its maximal emission peak at 465 nm makes it compatible with current BRET acceptors and its efficient application in two BRET-based assays has furthermore been shown. Its high activity allows lowering Donor amount needed to ensure sufficient BRET signal and may thus enhance the sensitivity of the method.

### BRET1 or BRET2?

Due to recent advances, the proper choice between BRET1 and 2 versions became difficult. Due to the lack of studies systematically comparing each BRET enhanced methods with each other, a ranking of the BRET signal and the amount of protein needed to reach it is hard to achieve. Both methods recently reached higher sensitivity, readout, and kinetics parameters that render them fully compatible with HTS. However, BRET1 basic method has been shown to be able to monitor PPI at endogenous expression level of proteins (Couturier and Jockers, 2003; Pfleger and Eidne, 2003) thus allowing the use of lower protein expression level than BRET2 in order to avoid active compound titration. Furthermore, Rluc and Rluc 8 as energy donor were systematically tested in BRET1 and BRET2 identical assays and showed the better sensitivity of BRET1 over BRET2 in living cells (Kocan et al., 2008). However, another study found the opposite (Dacres et al., 2009). To date, only BRET1-based P2I2 screening assays have been described and showed the feasibility of this approach (Mazars and Fåhraeus, 2010; Corbel et al., 2011). BRET1 seems to be nowadays the best suited BRET method to develop P2I2 screening assays until proven otherwise.

## How to Set Up a BRET Assay to Screen for PPI Inhibitors?

### Validation of the specificity of the interaction

The BRET signal is dependant on the ratio of donor/acceptor as it has been shown for years, using the well-known DSA, to show the specificity of the interaction. The first step to screen for P2I2 using BRET in cells is to verify this point by performing DSA experiments or other characterization such as untagged competitor protein cotransfection or effect of a known ligand promoting change of the BRET signal (Bacart et al., 2008; Ayoub and Pfleger, 2010).

### Production of inducible BRET cell lines

In order to set up a screening assay, the BRET signal has to be high, reproducible and stable, however, as revealed by DSA, fine changes in the donor or acceptor expression in transitory transfections will lead to a change in the BRET signal (Figure 2A). To ensure the stability and reproducibility of the signal needed for a screening assay, cell clones stably expressing the donor alone (Control cell line) and the donor/acceptor couple (BRET cell line) would be prepared as this was done for most BRET-based screening assays. Disrupting a PPI might be hard or quite worthy to achieve, this is why P2I2 screening assays developed until now were designed *in vitro* to allow compound tested to inhibit interaction before it takes place. For BRET-based assay, it is easily achieved if the studied interaction is naturally induced such as promoted receptor/effectors interaction upon ligand addition (Kamal et al., 2009; See et al., 2011). However, for constitutive interactions, designing such successful assays in living cell using BRET implies the use of a fast inducible system to add the chemical compound before inducing the target interaction (Corbel et al., 2011). Several mammalian tight inducible systems have been developed to reach this goal (Clackson, 1997). However, for screening protocol conveniences; repressed gene expression systems overcame by inducer molecule represents the best strategy. Several inducible systems are based on this scheme: Tet-on systems, based on a tet repressor (TetR) binding to tet operator elements of a promoter and displaced by addition of tetracycline derivatives thus allowing the target gene expression (Shockett and Schatz, 1996; Sun et al., 2007); Ecdysone systems and derivatives, based on glucocorticoids promoted association of an active steroid hormone nuclear receptor enabling expression of a target promoter (No et al., 1996; Xiao et al., 2003), and Q-mate^™^ based on a steric hindrance due to cumate repressor protein CymR bound to operator sites on the target promoter and which is released by addition of the inducer molecule cumate (Mullick et al., 2006). Two cell lines have to be developed to allow subtraction of the background BRET signal (from control cell line) from the interaction promoted BRET signal (BRET cell line). In order to gain comparable background luciferase activity in both cell lines, the BRET cell line would be advantageously prepared by introducing the acceptor tagged protein in the genome of the control cell line.

### Which BRET partner to induce?

Given the DSA curves, the maximal BRET signal is achieved when the donor is saturated by the acceptor. The resulting strategy would then be to express this one constitutively and the donor-fused partner in an inducible way. This kind of inverse DSA would lead to a high BRET signal tending to its maximal value as soon as the donor expression is induced. To ensure this ideal scenario, several parameters have to be taken into account when selecting the cellular clones. First of all, a low background expression of the donor is needed; otherwise a high BRET signal would be readily present before induction. Second, a sufficient acceptor expression has to be reached to ensure high maximal BRET values but low enough to avoid titration of compounds targeting this moiety. Third, during the induction process, the molecular amount of expressed donor would not exceed the one of the acceptor as the BRET signal would then decrease by free donor accumulation. Another important point to take into account is the location of the monitored interaction. Constitutively expressed acceptor would have reached its proper location whereas, upon induction, the donor will be neo-synthesized and a delay is then expected for it to reach final location and interact with its partners. The BRET signal appearance is then expected to be delayed, however, unless compound modify translation rate or transit through/between cellular compartments, this delay would be the same in presence or absence of screened compound incubation when verifying primary hits.

## How to Optimize a P2I2 BRET-Based Assay?

When setting up a primary screening assay, efforts have to be made to make it easy, fast, highly reproducible, and to lower the associated costs. To this aim, several parameters described below can be optimized when setting up P2I2 BRET-based screening assays to assume these efforts.

### Fast and easy protocol

**Figure 3:**
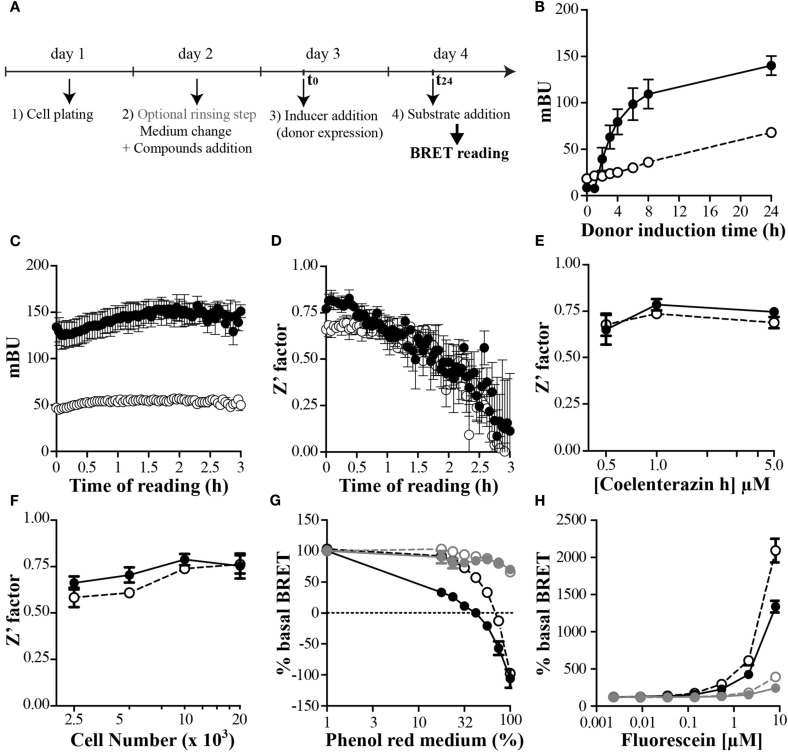
**Setting up and optimize a P2I2 BRET-based screening assay**. **(A)** Basic protocol of P2I2 BRET-based screening assay; **(B–H)** parameters analysis of the BRET signal monitored using OBRc/OBRGRP (●) and CD4/PLSCR1 (○) BRET-based screening assays, engineered using CHO-Trex cell lines to allow doxycyclin induction of donor. *n* = 3; **(B)** kinetic of the BRET induction: BRET signal monitored as a function of time after inducer addition (doxycyclin 0.1 μg/ml). **(C)** Kinetic of the BRET reading after coelenterazine h addition and **(D)** effect on the Z′ factor calculated from 8 points. *n* = 3. **(E)** Effect of substrate concentration or cell number used **(F)** on the Z′ factor (from 8 points). *n* = 3; **(G)** dose dependent effect of red phenol or Fluorescein **(H)** interfering compound in the medium when reading BRET. In gray when medium was removed before reading: OBRc/OBRGRP (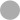) and CD4/PLSCR1 (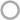).

The use of an inducible and stably expressing cell clones seems to be a prerequisite to ensure ease and reproducibility of such P2I2 BRET-based screening assays. An example using transitory transfection has shown that a known inhibitory compound was active in this assay (Mazars and Fåhraeus, 2010), however no hits based on this assay has been further published. On the contrary, a successful P2I2 screening assay using yeast stably expressing donor and acceptor respectively in an inducible and constitutive way has led to identification of chemical hits able to prevent the interaction between human cdk5 and p25 (Corbel et al., 2011). This study showed for the first time a real success for such P2I2 BRET-based screening assays. In order to keep the homogeneity of the test, efforts to set up a protocol avoiding unnecessary washing steps would be done. This can be achieved by some typical protocol as shown on Figure 3A: cells are first dispatched in wells, allowed to adhere, and rinsed (or not) to lower background donor expression. After this last step, addition of medium, compounds, inducer of the donor expression, and finally the donor substrate to perform the reading are then chronologically added.

### Kinetic of the monitored interaction

The use of an inducible system to ensure compound inhibitory action before the interaction takes place also lead to the problem of the kinetic of the studied interaction after induction. In order to know the maximal BRET value reachable as a function of induction time, a kinetic of the induction would be performed for each inducible P2I2 BRET-based assay developed. To show the feasibility of such an inducible BRET approach in mammalian cells, two cellular (tet-on based) inducible P2I2 BRET-based assays were developed to monitor the kinetic of the induction. The first test was based on a previous BRET demonstration that interaction of the leptin receptor (OBR) with OB-RGRP negatively regulated OBR expression at the cell surface and was implicated in leptin resistance (Couturier et al., 2007). The second, monitoring the interaction between CD4 and phospholipid scramblase 1 (PLSCR1) was developed, based on finding showing that disrupting this interaction may inhibit HIV entry into cells (Py et al., 2009). As seen on Figure 3B, a maximal BRET value was reached after 24–48 h of induction by doxycyclin for both assays and BRET signal was very stable. In order to shorten the screening campaign, a 24 h induction time would be chosen in the present cases.

### Kinetic of the BRET reading

When reading BRET, the kinetic of the *Rluc* emission is a crucial point as the BRET ratio is known to be stable only when it decreases. Depending of the temperature, the level of protein expressed, and of the developed test, the time to reach the decreasing activity step of the *Rluc* may vary from seconds to 5–15 min. The proper kinetic has then to be determined for each developed BRET assay and a corresponding delay has to be added in the process before reading. Another crucial point when performing a screening assay is the emitted light that have to last long enough to ensure at least the reading of an entire 96 or even 384 wells plate. However, it is well-known that the *Rluc* activity and *per se* the BRET signal cannot be monitored for a long time period. To date some BRET kinetic experiments have shown reliable signal for at least 30–60 min using coelenterazine h (Kocan et al., 2008; Matthiesen and Nielsen, 2011).

To circumvent the short lasting period of the Rluc emission and increase its light output, efforts have been made to develop some new substrates for BRET 2 or BRET 1 (Zhao et al., 2004). Two BRET1 compatible substrates have been produced by Promega to gain better kinetics parameters for *Rluc* *in vivo* and *in live* cells (ViViren and Enduren respectively). These substrates have protected oxidation sites to lower the autoluminescence due to their degradation and are metabolized to coelenterazine h by cellular esterases. The light output superiority over coelenterazine h has been shown for both these substrates (Otto-Duessel et al., 2006; Kimura et al., 2010), and the interest of using enduren was confirmed by studies showing maintained luciferase activity and BRET1 signal for up to 9 h (Dinh et al., 2005; Pfleger et al., 2006). However, the expensive cost of such substrates may explain their restricted use and hinder their application in BRET-based assay screening campaigns.

Using both our cellular inducible P2I2 BRET-based assays, we tested the kinetic of the BRET reading using common ceolenterazine h. Unexpectedly the BRET ratio remained reliable for as long as 3 h after substrate addition (Figure 3C) however, the *Z*′ value was compatible with screening (>0.5) for at least 80 min (Figure 3D). BRET monitored using coelenterazine h substrate is then sensitive enough and finally sufficiently long lasting to allow the automated addition of substrate in several plate and their reading over an extended time period using stackers.

### Influence of substrate concentration

The cost of a screening assay is a question of matter and regardless the price of compounds collection to be tested, a BRET-based assay includes the cost for the necessary substrate for each well to be read. To lower this cost, the total volume incubated in the wells has to be as low as possible to add the lower amount of substrate to reach the proper final concentration. Since the princeps publication describing BRET1 and until now, most BRET-based screening assays mostly used coelenterazine h at a final concentration of 5 μM (Boute et al., 2001; Charest et al., 2005; Hamdan et al., 2005; Laursen and Oxvig, 2005; Pfleger et al., 2006; Percherancier et al., 2009; Corbel et al., 2011; Kang et al., 2011) or even up to 30 μM (Vizoso Pinto et al., 2011). In order to monitor the effect of lowering the concentration of substrate on the BRET ratio and the *Z*′ parameter, both our P2I2 BRET-based assays were used. As shown on Figure 3E, the *Z*′ factor remain higher than 0.5 for a concentration of 1 μM and even 0.5 μM however it comes closer to the limit of 0.5. A final concentration of 1 μM can then be safely used when performing a P2I2 BRET-based screening campaign.

### Influence of the number of cells

When performing P2I2 screening assay, efforts have to be done to lower the amount of targeted complex to avoid or at least lower titration of the tested compound to gain high sensibility. The easy way to do it is to lower the number of plated cells, but the signal has to be still reliable and reproducible. To test this we plated varying number of cell from both our P2I2 assays we developed in 96 wells plate format and calculated the *Z*′ factor from the BRET results. As can be seen on the Figure 3F, the *Z*′ factor remains compatible with screening using lower cell number than 5000 but closest the 0.5 limit. 5000–10000 cells might then be used when performing such assay to ensure proper reliability.

### Influence of the BRET reading buffer assay

To perform a BRET assay in live cell, one would keep the cells in an as physiological context as possible and then perform the full experimental protocol from compound incubation to reading in proper cell culture medium. This is done currently in most studies, except for the final reading step which is mostly performed by replacing the medium with PBS containing the proper BRET substrate (See et al., 2011) or phenol red free medium. Indeed, when performing the BRET reading using medium containing red phenol, a shift in the BRET ratios is expected (Figure 3G), probably due to a change of the properties of the donor and/or the acceptor emissions or simply a physical change in the propagation of the light waves in the medium. Until recently, no dedicated study was done to monitor the effect of the reading buffer assay. Using a hGluc-(enterokinase cleavage site:-EYFP fusion, it has been shown that current buffers used to perform BRET reading such as Tris, Tricine, Sodium, HEPES, or MOPS are not the best to choose (Li et al., 2012). This study also showed that pH change of the medium promoted a change in the BRET signal (with a maximal value at ph 9), and furthermore that divalent cations such as Mg2+ and Ca2+ promoted a decrease in the BRET ratios. Most importantly, they have shown that adding imidazole to the reading medium promoted a 10-fold increase in the sensitivity of the assay and a sevenfold increase of the detection limit of the enterokinase activity. Although this was done using hGluc as donor, this study opens the way to monitor these parameters for other donors, as the monitored effects were not due to a drastic change in the luciferase activity but rather a change in the transfer efficiency. Future studies would find enhanced BRET buffers for BRET1 and BRET2 assays, in regards to the donor and acceptor used.

### Influence of colored and fluorescent compounds

As describe in the previous paragraph, the BRET signal can be modulated by the composition of the medium in which the reading is performed. Interfering compounds used in the reading buffer, on both control and BRET cell lines of a P2I2 BRET-based assay, would not be such a problem as the effect would be present in all wells measured leading to an overall increase or decrease of the signal. However, when performing a compound screening assay, if a compound in a particular well lead to such a change, a false positive or negative signal would be expected, as this well is compared to controls incubated with vehicle only. As shown using red phenol versus red phenol free medium (Figure 3G) a BRET decrease is monitored. A colored compound having such properties would be expected to lower the BRET signal due to a change in the medium properties, but not to a decrease in the studied interaction. On the other hand, fluorescent compounds sharing the same spectral properties than the acceptor, would also promote a change in the BRET signal, due to a saturation of the reading medium. A donor saturation effect leading to a BRET change would be expected by the free concentrated fluorescent compound if the donor emitted light overlaps the excitation one of this compound. In the case that the emission wavelength of the fluorescent compound is close to the acceptor emission, an artifactual BRET enhancement would be expected. Indeed, by incubating increasing concentration of Fluorescein on both our BRET screening assays, a huge BRET increase was monitored in a dose response manner. However, by replacing the medium containing Fluorescein by PBS before reading, the same BRET modulation was shown to be decreased by a 2 order dilution (Figure 3H) indicating that this effect was mostly mediated by the simple presence of Fluorescein in the medium. Therefore, when performing a P2I2 BRET-based screening assay, the reading of the fluorescence is necessary to exclude or to evidence those artifacts.

## What are the Expected Results?

**Figure 4:**
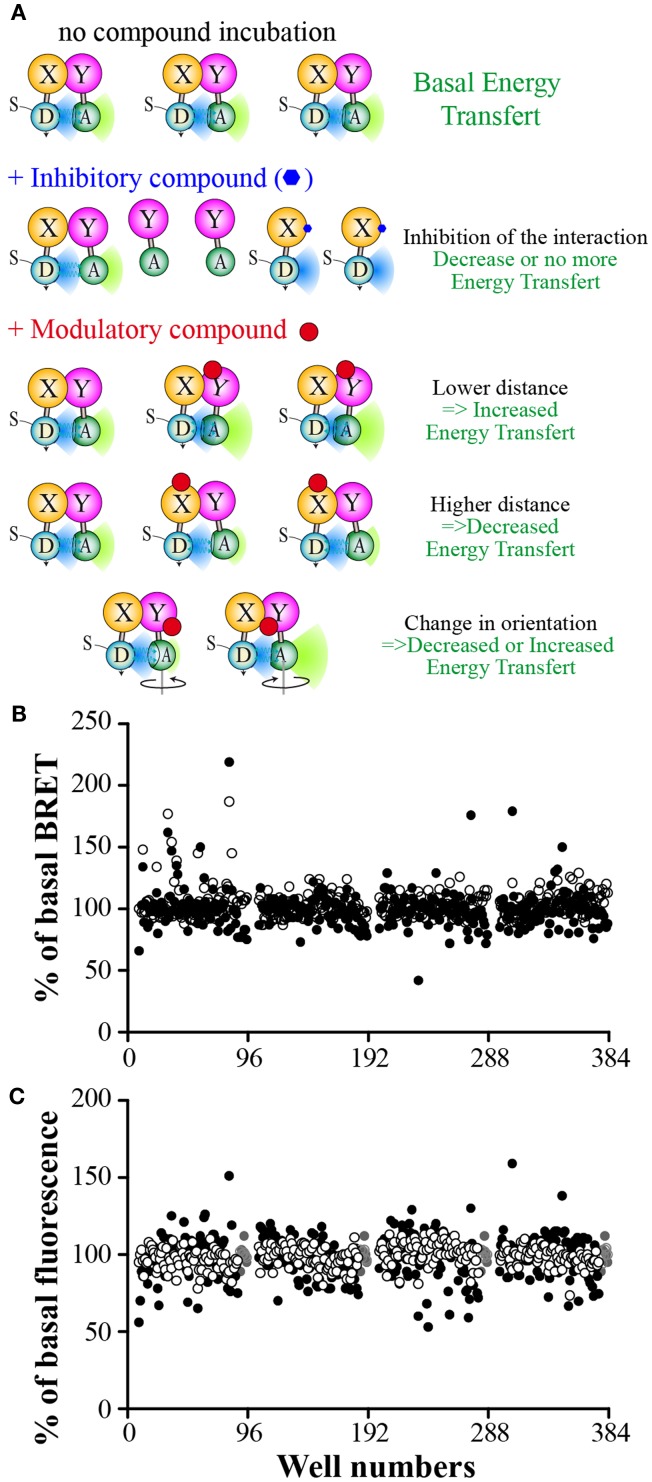
**Expected results from a P2I2 BRET-based screening assay**. **(A)** Different expected BRET change upon inhibitor or modulator compound action compared to basal BRET signal. *X* and *Y*: Protein *X* and *Y*; D: energy donor; A: energy Acceptor; S: BRET Substrate. **(B)** Results of a 320 compounds miniscreen using OBRc/OBRGRP (●) and CD4/PLSCR1 (○) BRET-based screening assays, expressed as% of basal BRET in absence of compound in each plate. **(C)** Fluorescence measured from the same plates as in **(B)** expressed as % of fluorescence value in absence of compound (represented by 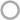).

If a molecule inhibits the studied interaction, a decrease in BRET signal is expected (Figure 4A). The BRET method is a well suited method for this purpose as the signal relies on the ratio of the two emitted wavelengths (respectively from the donor and the acceptor). The BRET intensity is then dependent of the percentage of interacting partners in the cell (Figure 2A). A PPI inhibitory compound is then expected to reduce the amount of the BRET interacting partners as well as increasing the non-interacting donor proportion, leading to an enhanced BRET signal decrease, higher than just decreasing the interacting partner amount. Such P2I2 screening using energy transfer methods, might then lead to lower the IC50 values, and therefore to enhance the detection limit of such active compound when using a given concentration.

As the energy transfer is dependent of the distance between donor and acceptor but also the relative orientation of their dipole moment (Stryer and Haugland, 1967; Hickerson et al., 2005; Majumdar et al., 2005), RET methods allows to monitor the presence of the targeted interaction as well as fast conformational changes in the studied complex (Vilardaga et al., 2003; Milligan, 2004; Lohse et al., 2008; Alvarez-Curto et al., 2010). Such conformational changes, prone to promote a RET signal change (increase or decrease), lead to expect a higher hit rate than other PPI monitoring methods. Hence, such conformational modulators are unable to be detected using classical methods basically monitoring the presence of the interaction, unless they also promote a dissociation of the targeted complex.

Among RET methods, BRET has been shown to allow the monitoring of intramolecular or intermolecular conformational changes with high sensitivity and even only tiny changes due to point mutations (Milligan, 2004; Bacart et al., 2008; Alvarez-Curto et al., 2010; Darbandi-Tehrani et al., 2010). P2I2 BRET-based screening assays might then detect interactions inhibitors but also conformational modulators (Figure 4A) that do not promote interaction disruption but might lead to a change in the biological function as well. BRET experiments have been successfully used to show ligands promoted conformational changes of receptors upon binding and leading to biological effects (Boute et al., 2001; Ayoub et al., 2002; Couturier and Jockers, 2003; Blanquart et al., 2006; Galés et al., 2006; Audet and Piñeyro, 2011). However, no systematic correlation between BRET increase or decrease and the biological effect is expected as agonists and antagonists were shown to promote a similar BRET change (Ayoub et al., 2002), no change (Terrillon et al., 2003), or even different BRET changes on same BRET assays (Elster et al., 2007), fully disrupting the correlation between the monitored signal and the expected biological effect.

Therefore, in PPI modulators BRET screening assays, if a known biological inducing control molecule is available, efforts would be focused on the design of a BRET assay able to monitor signal changes in presence of this compound. Nevertheless, compound promoting an opposite BRET change than the control used might represent another acting mechanism that could lead to a biological effect also.

To verify the feasibility of such a BRET approach to screen for P2I2 in mammal cells, we performed a miniscreens of 320 compounds using both our two PPI screening assays. As seen on Figure 4B, compounds were able to lower the BRET signal but also to increase it. Interestingly, some compounds were active on one assay but not the other. As expected, the total fluorescence reading (Figure 4C) showed that some compounds promoted changes in the overall fluorescence properties of the reading buffer in some wells. However, increased fluorescence was mild compared to those gained by Fluorescein but leading to no change in BRET signal (>10 and >2-fold increase respectively for OBRc/OBRGRP and CD4/PLSCR1; not shown). This indicates that these modifications prone to BRET signal increase might be of minor importance when performing P2I2 BRET-based screening assays, depending on the compound concentration used. On the contrary, BRET signal decrease, promoted by colored compounds might be more of concern as the decrease seen in the prescreen reached 50% of basal fluorescence, a change that promoted high BRET decrease when studying red phenol containing medium promoted BRET change (Figure 3G).

## Conclusion

Bioluminescence resonance energy transfer technique is well suited to set up high throughput P2I2 screening assays. It has several advantages over other methods: it is homogenous; it can be performed in live cells like FRET but with higher sensitivity; and allows the monitoring of the studied interactions in a whole intact cellular context. However, general guidelines have to be respected when setting up such assays. As in any BRET interaction monitoring, the specificity of this interaction has to be checked using classical DSA. Stable cells lines would be selected in order to assume ease and reproducibility of the assay and expression of the donor would be inducible to allow compound to inhibit the target interaction before it happens. Kinetic of the induction and interaction have then to be determined. The kinetic of the BRET signal reading and influence of substrate concentration has to be checked in order to choose the parameters leading to the best dynamic BRET output and highest *Z*′ factor value for the developed assay. Despite the fact that only one example of such a successful P2I2 screening assay (perfomed in yeast) has been published so far, this is a promising method to develop such assays in mammalian cells. One huge advantage of P2I2 BRET-based assay compared to classical methods is its ability to detect not only P2I2 but also conformational modulators of PPI, also able to promote the final biological targeted effect. A higher hit rate is then expected when using P2I2 BRET-based assays rather than with classical assays, only able to detect P2I2. Taken into account this huge advantage over other PPI monitoring techniques, its important optimization from the last years, and the still growing data of PPI leading to new potential drug target selection, a booming use of BRET to develop P2I2 assays would be expected in future years.

## Conflict of Interest Statement

The authors declare that the research was conducted in the absence of any commercial or financial relationships that could be construed as a potential conflict of interest.

## Appendix

### Engineering of OBRc/OBRGRP and CD4/PLSCR1 inducible BRET-based screening assays

#### Fusion protein expression vector cloning

Leptin receptor c isoform (OB-Rc) and phospholipide scramblase 1 (PLSCR1) were cloned in phase with Rluc8 coding sequence in pcDNA5 vector to gain pcDNA_5_-OBR-Rluc8 and pcDNA_5_-Rluc8-PLSCR1 fusion protein expressing vectors. Transcript Leptin receptor overlapping transcript 1 (OB-RGRP) and CD4 were cloned in pcDNA_3_ vector in phase with YPet.

#### Control and BRET cell lines engineering

Rluc8 fusion vectors were transfected in CHO-Trex (invitrogen) cell lines expressing a TetR and blasticidin antibiotic selection allowed to select resistant clones expressing luciferase activity. In both these BRET control cell line expressing the donor alone, the respective YPet-tagged partner was transfected and resistant clones selected using further G418 antibiotic selection to obtain BRET cell lines. OBRc-Rluc8/OBRGRP and CD4/PLSCR1 BRET cell line expressing the highest BRET signal upon stimulation by doxycyclin at 0.1 μg/ml for 24 h were selected. The use of the respective corresponding control cell lines allows to monitor BRET background to calculate mBRET from BRET cell lines.
